# Urban planning, noise pollution and mental health outcomes

**DOI:** 10.1192/j.eurpsy.2024.1397

**Published:** 2024-08-27

**Authors:** E. Abdelmoula, B. Abdelmoula, N. Bouayed Abdelmoula

**Affiliations:** ^1^LR AMC, Ecole Doctorale Sciences et Ingénierie Architecturales (ED-SIA), Tunis; ^2^Genomics of Signalopathies at the service of Precision Medicine - LR23ES07, Medical University of Sfax, Sfax, Tunisia

## Abstract

**Introduction:**

In large cities around the world, many sources of noise including traffic, domestic, construction, and industrial activities, contribute to urban noise pollution, which is now, a major concern in public health as declared by the WHO, for more than a decade (in 2011).

**Objectives:**

The aim of this study was to try to find potential recommendations and references in terms of urban planning, particularly with the emergence of smart cities, to combat the problem of noise pollution and related mental health hazards.

**Methods:**

We conducted a comprehensive review of the scientific literature using the following keywords: cities, smart cities, noise, pollution and mental health.

**Results:**

Our research found that the continuous exposure to high noise levels could lead to psychological and physiological problems, such as hearing disorders, high blood pressure, heart disease, inconvenience and sleep disorders. While recent evidence indicates that road traffic noise has a negative impact on mental health and that aircraft noise significantly increases the risk of depression, there are not enough studies to date to properly assess the relationship between urban noise pollution and mental health hazards such as anxiety, mood disorders, sexual disturbance, cognitive impairment, learning disabilities, dementia, etc. In the field of urban planning, there is also a lack of reliable data on individual exposure to environmental noise in space and time, and on its effects on mental health.

**Conclusions:**

Various noise mitigation strategies in urban renewal plans are proposed, such as the implementation of noise mapping to provide the detailed spatial distribution of noise levels in urban areas, their sources and time intervals, noise barriers along traffic arteries, vegetation and landscaping. New infrastructure projects involving new expressways and high-speed trains as well as the widening of major roads in their central areas are also suggested.

**Disclosure of Interest:**

None Declared

